# Recovery in gait and posture: a network-based approach to the assessment of rehabilitation effectiveness after spinal cord injury

**DOI:** 10.3389/fnetp.2026.1853254

**Published:** 2026-06-15

**Authors:** Tatyana Ageeva, Konstantin Grigarevichius, Aleksandr Sinitca, Davran Sabirov, Margarita Tsygankova, Nikita Pyko, Mikhail Bogachev, Albert Rizvanov, Yana Mukhamedshina

**Affiliations:** 1 Institute for Fundamental Medicine and Biology, Kazan Federal University, Kazan, Russia; 2 Department of Radio Engineering Systems, Saint Petersburg Electrotechnical University “LETI”, St. Petersburg, Russia; 3 Division of Medical and Biological Sciences, Tatarstan Academy of Sciences, Kazan, Russia; 4 Department of Histology, Cytology and Embryology, Kazan State Medical University, Kazan, Russia

**Keywords:** gait, network, posture, rehabilitation, spinal cord injury

## Abstract

Spinal cord injury (SCI) disrupts locomotion by affecting multiple physiological domains, including spinal conduction, reflex excitability, gait kinematics, and behavioral performance. Because recovery may be reflected not only in individual readouts but also in the organization of relationships among these readouts, we assessed post-SCI rehabilitation using a multi-domain correlation-network approach. Adult female Wistar rats underwent mild thoracic contusion SCI and were assigned to an untreated (SCI) or a treadmill-trained (SCI + TMT) group. Locomotor recovery was evaluated using treadmill testing, open-field walking, and the ladder rung test. Video recordings were processed using DeepLabCut/ALMA-derived kinematic variables and were combined with behavioral scores, electrophysiological measures, and morphometric assessment. Group differences in kinematic parameters were evaluated using Mann–Whitney U tests with Benjamini–Hochberg false-discovery-rate correction, and correlation-network graphs were constructed from Spearman associations between selected electrophysiological and kinematic indicators. At 28 days post-injury (dpi), treadmill rehabilitation was associated with partial normalization of open-field stride-time metrics: median stride time recovered by 83%–84% toward the intact baseline for hind paws and by 81% for knee points relative to the SCI–intact difference, while stride-time variability normalized by 92%–100% for knee points. The hindlimb-to-forelimb stride-time ratio was closer to the intact value in SCI + TMT than in untreated SCI animals (1.03 vs. 1.25; intact: 1.00). Correlation-network analysis after multiple-comparison correction identified a limited set of associations involving bilateral H-wave amplitude, contralateral H-wave latency–hindlimb gait timing, and hindlimb/knee stride-interval indicators in the trained group. These findings suggest that treadmill rehabilitation after SCI is reflected not only in individual locomotor metrics but also in the organization of cross-domain statistical associations.

## 1 Introduction

Spinal cord injury disrupts the transmission of descending motor commands from the brain to spinal circuits involved in locomotion, leading to severe impairments in walking and frequently resulting in paralysis (Lorach et al., 2023; Ahuja et al., 2017; Bickenbach et al., 2013). Importantly, although the lumbar and sacral spinal neurons responsible for generating locomotor activity are often preserved, the interruption of the descending pathways deprives these circuits of the supraspinal input required for effective walking (Courtine et al., 2008; Courtine and Sofroniew, 2019). Locomotor behavior is inherently rhythmic and requires coordinated activation of multiple muscles, being largely organized by spinal central pattern generators (CPGs) that integrate descending brain signals with somatosensory feedback (Su et al., 2025; Grillner, 2011; Frigon, 2017). Disruption of this multilevel control due to spinal cord injury therefore leads to impaired automatic gait regulation, manifesting as reduced locomotor speed and altered locomotor patterns in both humans and animal models (Danner et al., 2023; Minassian et al., 2023). This complexity places specific demands on the way locomotor recovery after spinal cord injury is assessed. Traditional behavioral scoring scales, including the Basso, Beattie, and Bresnahan (BBB) scale and ladder walking tests, are widely used in preclinical spinal cord injury research. However, their ordinal and coarse nature limits sensitivity to subtle alterations in gait structure and interlimb coordination, and such approaches may therefore fail to capture nuanced locomotor deficits that persist after injury (Krizsan-Agbas et al., 2014; Su et al., 2025). Open-field locomotor scoring provides limited information on postural control and step timing, both of which are essential for automatic gait regulation (Krizsan-Agbas et al., 2014). Clinical studies further indicate that sensor-derived gait measures, including indices of variability and compensatory movement patterns, complement conventional walking tests by revealing aspects of functional gait that are not reflected by standard clinical assessments (Willi et al., 2024; Sung and Park, 2024). The absence of standardized and objective gait metrics across preclinical models hampers comparisons between studies and treatment strategies, motivating the adoption of data-driven, multi-feature gait analysis frameworks (Aljovic et al., 2022; Werner et al., 2023). Deep learning–based methods improve the accuracy and objectivity of behavioral tracking compared to traditional approaches, allowing quantitative analysis of locomotion from video data (Sturman et al., 2020; Sato et al., 2022). Markerless pose estimation methods such as DeepLabCut enable the accurate extraction of body kinematics from video data (Mathis et al., 2018). Based on these outputs, downstream frameworks, including Automated Ladder Movement Analysis (ALMA), provide quantitative analysis of gait structure, paw placement, and interlimb coordination during treadmill and overground locomotion (Aljovic et al., 2022).

Previously, we have analyzed the complexity of gait structure and dynamics and their disturbances affected by Alzheimer’s disease in an animal model focusing on an open field arena test protocol and a coordinated random walking model perspective. Our data indicated significant alterations in motion patterns in the pathophysiological group associated with an early onset of Alzheimer’s disease compared to control animals that were explicitly reflected by maximum full and partial cross-correlations and associated delays between movement patterns exhibited by different paws, in turn represented by a graphical model extracted by the modified detrended partial cross-correlation analysis (DPCCA) in an automated pipeline design (Bogachev et al., 2023).

The remainder of the paper is organized as follows. The next Section 2 contains a description of the data acquisition and processing pipeline, Section 3 presents numerical results, and Section 4 presents a discussion of the key findings of the study.

## 2 Materials and methods

### 2.1 Animal study design

**FIGURE 1:**
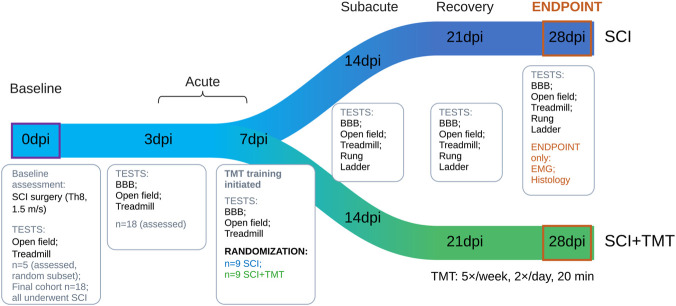
Experimental design and rehabilitation timeline. Adult female Wistar rats underwent baseline assessment before mild thoracic contusion SCI at the vertebral Th8 level on day 0. A randomly selected subset was used to represent intact baseline values (n = 5). After early post-injury assessment at 3 and 7 days post-injury (dpi), animals were randomized into untreated SCI (n = 9) and treadmill-trained SCI + TMT (n = 9) groups. Treadmill training (TMT) began at 7 dpi and was performed 5 days per week for 3 weeks, twice daily for 20 min. Behavioral and kinematic assessments included BBB scoring, open-field walking, treadmill testing, and the ladder rung test at the indicated time points. Electrophysiological recording and histological analysis were performed at the endpoint. Abbreviations: BBB, Basso–Beattie–Bresnahan locomotor rating scale; dpi, days post-injury; EMG, electromyography; SCI, spinal cord injury; TMT, treadmill training.

The overall experimental design and rehabilitation timeline are summarized in Figure 1.

#### 2.1.1 Animal

Adult female Wistar rats (weight 250–300 g; Krolinfo Ltd., Russia) were used in the experiments. The approval for the animal study was obtained from the local ethics committee of Kazan (Volga region) Federal University (Approval No. 50, dated 26 September 2024). The rats were housed in standard transparent plastic cages under controlled environmental conditions (temperature $23\pm 1^{{}^{\circ}}C$ , 60%–65% humidity, 12-h light/dark cycle) with *ad libitum* access to food and water.

#### 2.1.2 Spinal cord injury

All surgical procedures were performed under anesthesia induced with Zoletil (20 mg/kg, intramuscularly; Virbac) after premedication with xylazine hydrochloride (10 mg/kg, intramuscularly; Nita-Pharm, Russia). After laminectomy at the vertebral Th8 level, rats were subjected to a mild contusion SCI (1.5 m/s) using an Impact One Stereotaxic Impactor (Leica). After surgery, animals were administered daily intramuscular enrofloxacin (10 mg/kg; Novakorm) for 7 days and underwent manual bladder expression twice daily until the return of spontaneous bladder function.

The final analyzed cohort consisted of 18 adult female Wistar rats. Baseline (intact) motor function was assessed prior to surgery (day 0) in a randomly selected subset of animals (n = 5, Intact) from this cohort. Seven days after SCI, animals were randomly assigned to one of two experimental groups: non-trained rats that underwent SCI only (n = 9, SCI) and rats with the same injury that additionally received treadmill training starting on day 7 post-injury (n = 9, SCI + TMT).

#### 2.1.3 Time-series (movement) features

The first part of the analyzed feature set includes data obtained from three types of tests: open field, treadmill, and ladder rung tests. For all kinds of tests we recorded video using a Canon EOS 250D kit camera (EF-S 18–55 mm f/4–5.6 IS STM) at a resolution of 1920×1080 pixels and a frame rate of 60 frames per second with standard IPB compression. For all tests except the ladder rung test, videos were processed using DeepLabCut as described in Section 2.2.

##### 2.1.3.1 Treadmill training and test

The treadmill training was performed using an IITC Life Science Treadmill for Mice and Rats (800 Series Treadmill). Physical exercise was initiated on day 7 after SCI and was carried out 5 days per week for 3 weeks. The training sessions lasted 20 min and were conducted twice daily with an interval of 2 h. The treadmill speed ranged from 6 cm/s (3.6 m/min) to 21 cm/s (12.6 m/min), depending on their functional status, with a gradual increase in workload.

**FIGURE 2:**
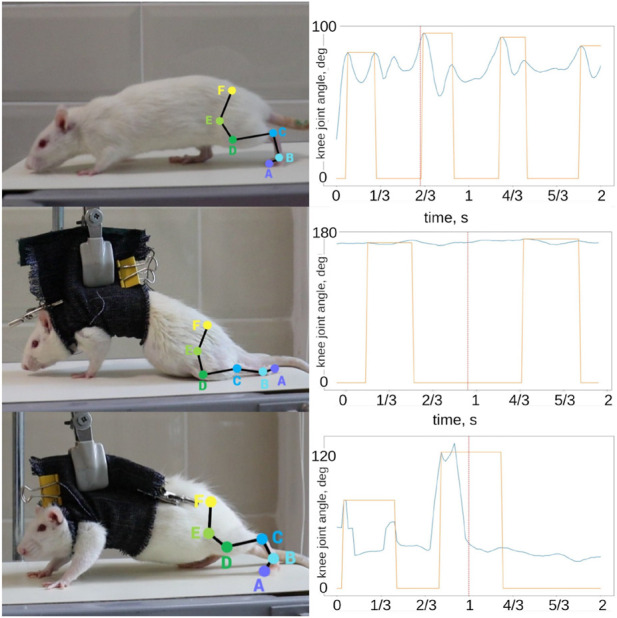
Visualization of detected joint points comprising the skeleton model. Blue curves exemplify knee joint angle dynamics, while orange lines indicate the detected stride cycles. Dashed red line indicates the time position corresponding to the snapshot to the left of the plot.

For the treadmill test, we adopted an even more detailed configuration comprising 13 anatomical landmarks: snout, tail base, body center, four paws, left and right knee joints, left and right shoulder joints, and two additional points along the spine (Figure 2). This high-resolution skeletal representation allowed the precise characterization of the gait cycle phases, joint kinematics, and interlimb coordination under steady-state locomotion. In contrast, for the ladder rung test, tracking only the four paws was sufficient, as the primary analytical goal was to detect foot-slip errors and assess asymmetries in paw placement—tasks that did not require full-body posture reconstruction.

##### 2.1.3.2 Ladder rung test

Preoperative habituation to the ladder rung apparatus (OpenScience, Russia) was conducted for 3 days. Behavioral tests were initiated 7 days post-injury (dpi) and performed twice weekly until 28 dpi.

The rats completed an average of 4 complete ladder traverses in alternating directions during the 3-min observation period. Limb placement was evaluated based on paw positioning on the rungs and the occurrence of limb slippage between rungs in the event of a misstep. During video analysis, a step was scored when movement in 3 joints was observed ( ≥4 BBB points) or during full plantar paw placement ( ≥9 BBB points) (Metz and Whishaw, 2009). This modification of the standard rung ladder protocol allows for a more sensitive assessment of locomotor function in injured animals by accounting for all limb movements, including those not yet achieving consistent plantar support. Quantitative analysis was performed across 4 traverses per animal and included the following parameters: (1) total number of steps; (2) number of errors (Zhang et al., 2016); and (3) traversal score for each pass (Metz and Whishaw, 2009).

Quantitative gait analysis in the ladder rung test was performed using the specialized software ALMA (Automated Ladder Movement Analysis). This platform automatically extracts a comprehensive set of spatiotemporal and kinematic features, including foot-slip frequency, slip depth relative to the rung, stance and swing phase durations for each limb, interlimb coordination metrics (e.g., step regularity index), left–right asymmetry, and precise trajectories of wrist and ankle joints. By combining high-accuracy pose estimation with movement classification algorithms, ALMA provides an objective and reproducible assessment of motor function during complex, goal-directed locomotion.

##### 2.1.3.3 Open-field walking test

For detailed gait analysis, the animals were tested in an open-field apparatus consisting of an arena measuring 100.0×50.0×10.0 cm with a transparent PVC floor. To allow adaptation to the experimental conditions, rats were placed in the arena for 3 days prior to surgery. Behavioral testing was conducted on postoperative days 3, 7, 14, 21, and 28 in both experimental groups. In addition, to determine the intact (baseline) level of motor function, animals were also tested prior to surgery (day 0).

During the open-field test the camera lens was positioned beneath the transparent floor of the open-field arena to enable ventral-view recordings. Recording was performed under normal ambient lighting conditions to avoid potential effects of artificial illumination on animal behavior.

In the open field test, we initially employed a DeepLabCut model tracking seven anatomical points: snout, tail base, body center, and the four paws reported in our previous work (Bogachev et al., 2023). However, qualitative and quantitative evaluation of tracking performance revealed insufficient precision in reconstructing biomechanically relevant parameters, particularly during partial occlusions and rapid directional changes. To address this limitation, we modified the model to nine keypoints additionally incorporating the left and right knee joints. This enhancement significantly improved the estimation of limb flexion angles and increased the temporal stability of trajectory reconstruction, which proved essential for analyzing spontaneous and irregular locomotion patterns.

#### 2.1.4 Scalar features and measurements

##### 2.1.4.1 BBB score

Voluntary locomotor activity was assessed using the BBB locomotor rating scale by two independent observers blinded to the experimental groups. Preoperative habituation to the open-field environment was conducted for 3 days. Starting on day 7 post-SCI, locomotor activity was evaluated twice a week at a consistent time of day by placing each rat individually in the center of the open-field arena and scoring locomotor parameters during a 3-min observation period. The final BBB scores were obtained as the average of the two independent evaluations.

##### 2.1.4.2 Electromyography

Stimulated electromyography was conducted using an 8-channel Neuro-MVP-8 electromyography system (Neurosoft, Russia). M and H-waves, somatosensory evoked potentials (SEPs), and motor evoked potentials (MEPs) were recorded in response to sciatic nerve stimulation.

##### 2.1.4.3 Morphometric analysis

At 28 dpi, the rats were anesthetized and subjected to intracardiac perfusion with 10% neutral buffered formalin (Biovitrum, Russia). The spinal cord segment extending from 5 mm rostral to 2.5 mm caudal to the injury epicenter was additionally fixed in 10% formalin at room temperature overnight, followed by incubation in 30% sucrose solution. After embedding in Tissue-Tek O.C.T. Compound (Sakura), 20- μ m-thick longitudinal sections of the spinal cord were prepared using a cryostat FS800A (RWD Life Science, China). The area of pathological cavities was quantified morphometrically using NIH ImageJ software (Schneider et al., 2012; Rueden et al., 2017).

### 2.2 Video processing and time series postprocessing

To quantitatively assess locomotor behavior in laboratory rats across three distinct behavioral paradigms: treadmill walking, open-field exploration, and the ladder rung coordination test, we developed three independent pose estimation models based on the ResNet50 (He et al., 2016) architecture within the DeepLabCut framework. Each model was trained exclusively on video data corresponding to its specific experimental setup, taking into account variations in camera perspective, lighting conditions, movement velocity, and spatial scene configuration. This task-specific training strategy optimized the detection accuracy of key anatomical landmarks (for example, snout, four paws, tail base, and body center) under protocol-specific constraints and enabled robust reconstruction of 2D movement trajectories.

However, when analyzing the trajectories of the treadmill and open field tests, conventional signal preprocessing techniques, including spectral analysis, autocorrelation, and standard low/high-pass filters, proved inadequate. This was due to the high non-stationarity of biomechanical signals, tracking artifacts, and substantial inter-step variability inherent in rodent locomotion. Traditional approaches to estimating step frequency and amplitude yielded unstable results during speed transitions or brief pauses, which are common in spontaneous behavior.

To overcome these challenges, we developed a custom post-processing algorithm that integrates robust filtering based on the interquartile range (IQR) to suppress outliers and a variable-length moving average window dynamically adapted to local motion dynamics. This hybrid approach effectively removes the noise caused by keypoint detection errors while preserving the temporal fidelity of gait phases. Using cleaned trajectories, the algorithm automatically identifies paw contact and lift-off events and subsequently computes all required parameters.

For the treadmill test, the metrics extracted include the total step count, the average duration of the step (s), the average maximum and minimum joint flexion angles and the average step height (vertical displacement during the swing phase). For the open field test, the algorithm performs a limb-specific gait characterization for each tracked point (forepaws, hindpaws, knees), computing for each the median, mean, coefficient of variation (Kvar) and fraction of zero intervals (Frac) for both stride time (time between consecutive contacts of the same paw) and stride interval (temporal spacing between steps of different limbs).

For open-field median-based parameters, values are presented as group mean ± SEM of the per-animal median values.

### 2.3 Correlation-network reconstruction of cross-domain indicators

To assess cross-domain statistical relationships among rehabilitation-related readouts, we constructed undirected weighted correlation networks separately for each experimental group and time point. Each node represented one pre-specified scalar indicator. The network included electrophysiological indicators, namely, left and right H-wave amplitude and latency, and kinematic indicators derived from open-field recordings, namely, median stride interval for the left front paw (LFP), right front paw (RFP), left hind paw (LHP), right hind paw (RHP), left knee (LK), and right knee (RK). Cross-domain electrophysiology–kinematics networks were constructed and interpreted only for group/time-point combinations for which both electrophysiological and kinematic indicators were available. M-wave amplitude and latency were analyzed analogously as peripheral neuromuscular electrophysiological indicators and used as an internal comparison for the H-wave-based network findings.

For each group, all pairwise associations between nodes were calculated using Spearman’s rank correlation coefficient $\left(r_{s}\right)$ . Edge weight was defined as the signed Spearman coefficient. Positive edges therefore indicate that larger values of two indicators tend to co-occur across animals, whereas negative edges indicate inverse rank relationships. For visualization only, edges with $|r_{s}|<0.30$ were omitted to reduce visual clutter; this threshold was not used as a criterion of statistical significance.

Between-group differences in individual edge weights were assessed using Fisher’s z transformation. Because Fisher’s test assumes Pearson correlations, Spearman coefficients were first converted to Pearson-equivalent coefficients as $\rho =2\sin\left(\pi r_{s}/6\right)$ . Fisher’s transformation was then applied as z=0.5⁡ln[(1+ρ)/(1−ρ)] . For each edge, the between-group statistic was calculated as $Z=\left(z_{1}-z_{2}\right)/\sqrt{1/\left(n_{1}-3\right)+1/\left(n_{2}-3\right)}$ , where $n_{1}$ and $n_{2}$ are the numbers of animals in the compared groups. Two-sided p-values were obtained from the standard normal distribution and adjusted for multiple edge-wise comparisons using the Benjamini–Hochberg false-discovery-rate procedure. Adjusted values are reported as $p_{\text{adj}}$ . Network-level findings were interpreted as corrected statistical association patterns rather than as evidence of causal physiological interactions.

### 2.4 Statistical analysis

**FIGURE 3:**
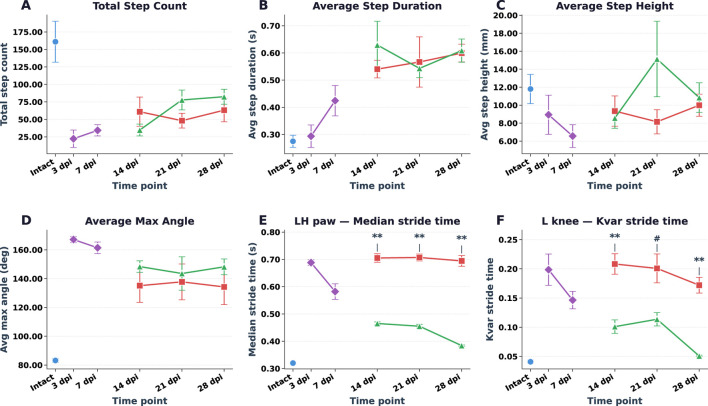
Gait and postural parameters across intact and post-SCI time points. Values are group mean ± SEM of per-animal values. **(A)** Total step count, **(B)** average step duration, **(C)** average step height, **(D)** average maximum joint angle, **(E)** median stride time (left hind paw, open field), **(F)** coefficient of variation of stride time (left knee, open field). Markers in **(E,F)** denote SCI vs. SCI + TMT comparisons at 14, 21 and 28 dpi: ** $p_{\text{adj}}<0.01$ ; # $p_{\text{adj}}<0.10$ (trend). **(A–D)** were not significant after correction.

Kinematic parameters (Figure 3) were analysed at the level of individual animals. For each time point, the SCI and SCI + TMT groups were compared using the two-sided Mann–Whitney U test, chosen because the number of valid observations varied across parameters and time points, normality could not be reliably assessed in these relatively small samples, and several gait metrics (stride-time variability, step height) are known to deviate from Gaussianity. Effect sizes are reported as Cliff’s δ , where |δ|≥0.474 indicates a large effect. To control the false discovery rate across the family of primary post-randomization comparisons (six parameters × three time points: 14, 21, 28 dpi), p -values were adjusted using the Benjamini–Hochberg procedure; adjusted values are denoted $p_{\text{adj}}$ . As a secondary analysis, each group at the final time point (28 dpi) was compared with the intact baseline using the same test, with Benjamini-Hochberg false-discovery-rate correction (BH-FDR) applied within that family. Significance in figures is marked as ** $p_{\text{adj}}<0.01$ and # $0.05\leq p_{\text{adj}}<0.10$ (statistical trend). Between-group comparisons of correlation-network edge weights were performed using Fisher’s z transformation as described in Section 2.3, with Benjamini–Hochberg false-discovery-rate correction for multiple edge-wise comparisons. All analyses were performed in Python 3.11 (scipy.stats, numpy).

## 3 Results

### 3.1 Total step count

At baseline, intact animals performed 161.00 ± 29.27 steps per session (Figure 3A). At 3 dpi, the combined post-SCI cohort showed 22.28 ± 12.44 steps, with 4 of 18 animals producing no detectable steps. At 7 dpi, the step count was 34.32 ± 8.01. At 14 dpi, step counts remained variable. At later time points, the SCI + TMT group showed higher step counts than the SCI group: 77.75 ± 14.13 vs. 48.12 ± 10.61 at 21 dpi and 82.38 ± 10.70 vs. 63.12 ± 16.62 at 28 dpi, corresponding to 51% and 39% of the intact baseline, respectively. The SCI group exhibited greater inter-animal variability throughout the observation period.

### 3.2 Average step duration

Intact step duration was 0.27 ± 0.02 s (Figure 3B). The combined post-SCI cohort showed 0.29 ± 0.04 s at 3 dpi and 0.42 ± 0.06 s at 7 dpi. From 14 dpi, step duration was comparable between groups at all time points: 0.54 ± 0.03 vs. 0.63 ± 0.09 s at 14 dpi, 0.57 ± 0.09 vs. 0.54 ± 0.03 s at 21 dpi, and 0.60 ± 0.03 vs. 0.61 ± 0.04 s at 28 dpi (SCI and SCI + TMT, respectively). At 28 dpi, step duration in both groups was 2.2-fold higher than at baseline.

### 3.3 Average step height

Step height in intact animals was 11.80 ± 1.63 (Figure 3C). The combined post-SCI cohort showed 8.93 ± 2.17 at 3 dpi (76% of baseline) and 6.56 ± 1.26 at 7 dpi (56%). The SCI and SCI + TMT groups were comparable at 14 dpi (9.34 ± 1.69 and 8.56 ± 1.14, respectively). At 21 dpi, the SCI + TMT group reached 15.13 ± 4.20, exceeding the baseline level, while the SCI group was at 8.15 ± 1.35. By 28 dpi, both groups were similar: 9.99 ± 1.23 (SCI, 85% of baseline) and 10.84 ± 1.65 (SCI + TMT, 92%).

### 3.4 Hindlimb joint angle kinematics

In intact animals, the mean maximum joint angle was 83.18 ± 1.48° and the minimum angle 58.67 ± 3.27°, yielding a range of motion (ROM) of 24.5° (Figure 3D). At 3 dpi, both angles shifted toward full extension: maximum 167.16 ± 2.14°, minimum 160.20 ± 4.96°, ROM 7.0°. At 7 dpi, the maximum was 161.41 ± 4.02°, minimum 151.62 ± 4.92°, ROM 9.8°. The maximum angle was lower in the SCI group than in the SCI + TMT group from 14 dpi onward: 135.12 ± 11.62° vs. 148.35 ± 4.00° at 14 dpi, 137.76 ± 12.44° vs. 143.57 ± 11.58° at 21 dpi, and 134.24 ± 12.31° vs. 148.19 ± 5.45° at 28 dpi. A similar pattern was observed for the minimum angle: SCI values were 119.69 ± 13.72°, 115.70 ± 13.88°, and 107.68 ± 14.76° at 14, 21, and 28 dpi, respectively; SCI + TMT values were 137.03 ± 5.16°, 116.47 ± 14.70°, and 120.34 ± 6.83°. Both groups remained above the intact baseline at all time points. By 28 dpi, ROM was 26.6° (SCI) and 27.8° (SCI + TMT), comparable to the intact value of 24.5°.

### 3.5 Open field median stride time and variability

At baseline, left hind paw median stride time was 0.32±<0.01 s (Figure 3E). At 3 dpi, the combined post-SCI cohort showed 0.69 ± 0.01 s, and at 7 dpi the value was 0.58 ± 0.03 s. From 14 dpi, the SCI + TMT group showed lower values than the SCI group at all time points: 0.47 ± 0.01 vs. 0.71 ± 0.02 s at 14 dpi, 0.46 ± 0.01 vs. 0.71 ± 0.01 s at 21 dpi, and 0.38 ± <0.01 vs. 0.69 ± 0.02 s at 28 dpi, corresponding to 120% and 217% of the intact baseline, respectively. At 28 dpi, the right hind paw showed similar values: 0.38 ± <0.01 vs. 0.69 ± 0.01 s in SCI + TMT and SCI, respectively.

At baseline, left knee median stride time was 0.32 ± <0.01 s. At 3 dpi, the combined post-SCI cohort showed 0.70 ± 0.01 s, and at 7 dpi the value was 0.58 ± 0.03 s. From 14 dpi, the SCI + TMT group showed lower values than the SCI group at all time points: 0.45 ± 0.01 vs. 0.68 ± 0.01 s at 14 dpi, 0.47 ± <0.01 vs. 0.67 ± 0.01 s at 21 dpi, and 0.39 ± <0.01 vs. 0.70 ± 0.01 s at 28 dpi, corresponding to 123% and 220% of the intact baseline, respectively. At 28 dpi, the right knee showed similar values: 0.39 ± <0.01 vs. 0.69 ± 0.02 s in SCI + TMT and SCI, respectively.

At baseline, left front paw median stride time was 0.32 ± <0.01 s. At 3 dpi, the combined post-SCI cohort showed 0.58 ± 0.01 s, and at 7 dpi the value was 0.49 ± 0.02 s. From 14 dpi, the SCI + TMT group showed lower values than the SCI group at all time points: 0.43 ± 0.01 vs. 0.55 ± 0.01 s at 14 dpi, 0.43 ± 0.01 vs. 0.55 ± 0.01 s at 21 dpi, and 0.37 ± <0.01 vs. 0.56 ± 0.01 s at 28 dpi, corresponding to 116% and 173% of the intact baseline, respectively.

The hindlimb-to-forelimb stride time ratio (left hind paw/left front paw median stride time) was 1.00 in intact animals and 1.20 at 3 dpi. At 28 dpi, the ratio was 1.25 in SCI and 1.03 in SCI + TMT.

At baseline, left knee Kvar stride time was 0.04 ± <0.01 (Figure 3F). At 3 dpi, the combined post-SCI cohort showed 0.20 ± 0.03, and at 7 dpi the value was 0.15 ± 0.02. From 14 dpi, the SCI + TMT group showed lower values than the SCI group at all time points: 0.10 ± 0.01 vs. 0.21 ± 0.02 at 14 dpi, 0.11 ± 0.01 vs. 0.20 ± 0.03 at 21 dpi, and 0.05 ± <0.01 vs. 0.17 ± 0.01 at 28 dpi, corresponding to 124% and 420% of the intact baseline, respectively. At 28 dpi, the right knee showed similar values: 0.05 ± <0.01 vs. 0.18 ± 0.03 in SCI + TMT and SCI, respectively.

At 28 dpi, left hind paw Kvar stride time was 0.05 ± <0.01 in SCI + TMT and 0.19 ± 0.02 in SCI (intact baseline: 0.03 ± <0.01 ); right hind paw Kvar was 0.04 ± <0.01 vs. 0.18 ± 0.02, respectively (intact: 0.04 ± <0.01 ).

Relative to the difference between the SCI and intact groups at 28 dpi, the SCI + TMT group showed 83% normalization for left hind paw median stride time, 84% for right hind paw, 81% for left knee, and 81% for right knee. For Kvar stride time, normalization was more complete: 92% for left knee and 100% for right knee.

### 3.6 Ladder rung performance

The ladder rung test was used as a complementary assessment of skilled locomotion and paw placement during goal-directed walking. At 3 and 7 dpi, no completed ladder steps were detected in the combined post-SCI cohort. At 14 dpi, the SCI + TMT group showed a higher number of completed ladder steps than untreated SCI animals (4.44 ± 1.18 vs. 0.95 ± 0.95). At 21 dpi, the corresponding values were 8.00 ± 0.66 in SCI + TMT and 7.31 ± 1.34 in SCI. At 28 dpi, ladder performance was broadly comparable between groups (7.75 ± 0.55 in SCI + TMT vs. 9.14 ± 1.19 in SCI). Because ladder rung outcomes were used as complementary behavioral measures, they were not included as nodes in the main correlation-network reconstruction.

### 3.7 Correlation-network analysis of cross-domain associations

**FIGURE 4:**
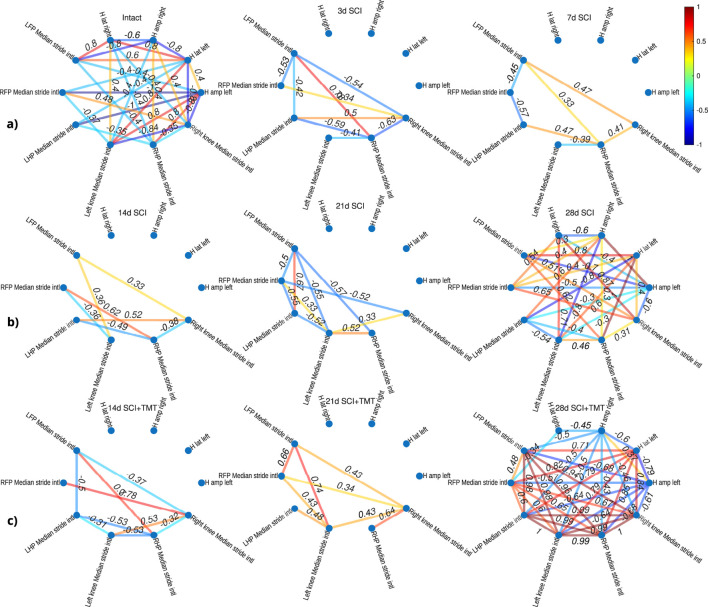
Correlation-network visualization of selected electrophysiological and kinematic indicators across the experimental timeline. **(a)** Intact baseline and early post-SCI networks at 3 and 7 dpi. **(b)** Untreated SCI networks at 14, 21, and 28 dpi. **(c)** SCI + TMT networks at 14, 21, and 28 dpi. Nodes represent pre-specified scalar indicators: H amp left/right, H-wave amplitude recorded from the left/right side; H lat left/right, H-wave latency; LFP/RFP, left/right front paw; LHP/RHP, left/right hind paw; LK/RK, left/right knee. Gait nodes show median stride interval derived from open-field recordings. Edges represent signed Spearman rank correlation coefficients $\left(r_{s}\right)$ between node pairs within the corresponding group/time point. Edge labels indicate $r_{s}$ values; edge color indicates the sign of the association, and edge thickness indicates the absolute magnitude of $r_{s}$ . Edges with $|r_{s}|<0.30$ were omitted for visualization only to reduce visual clutter; this threshold was not used as a criterion of statistical significance. Positive associations indicate that larger values of two indicators tend to co-occur across animals, whereas negative associations indicate inverse rank relationships. Between-group edge-wise differences were tested using Fisher’s z transformation with Benjamini–Hochberg false-discovery-rate correction.

Figure 4 summarizes signed Spearman correlation networks constructed from selected electrophysiological and kinematic indicators. Edge-wise between-group differences were assessed using Fisher’s z transformation followed by Benjamini–Hochberg false-discovery-rate correction, as described in Section 2.3.

After correction, the endpoint SCI vs. SCI + TMT comparison retained a limited set of edge-wise differences. These included a bilateral association between left and right H-wave amplitudes (H amp left–H amp right, $p_{\text{adj}}=0.001$ ) and one cross-domain association between left H-wave latency and the right hind paw median stride interval (H lat left–RHP median stride interval, $p_{\text{adj}}=0.001$ ). Additional corrected differences were concentrated mainly among hindlimb and knee stride-interval indicators: LFP–LHP $\left(p_{\text{adj}}=0.010\right)$ , LFP–left knee $\left(p_{\text{adj}}=0.005\right)$ , LHP–left knee $\left(p_{\text{adj}}=0.001\right)$ , LHP–RHP $\left(p_{\text{adj}}=0.001\right)$ , LHP–right knee $\left(p_{\text{adj}}=0.005\right)$ , left knee–RHP $\left(p_{\text{adj}}=0.005\right)$ , left knee–right knee $\left(p_{\text{adj}}=0.005\right)$ , and RHP–right knee $\left(p_{\text{adj}}=0.001\right)$ . No corrected RFP-related association was detected, and no association involving M-wave amplitude or latency survived correction.

Thus, after correction for multiple comparisons, the network results are interpreted as a sparse pattern of corrected statistical associations. The retained links were mainly concentrated in bilateral H-wave amplitude, one contralateral H-wave latency–hindlimb gait association, and hindlimb/knee stride-interval relationships.

## 4 Discussion

The central finding of this study is that treadmill rehabilitation after spinal cord injury was associated not only with changes in individual locomotor metrics, but also with a more organized pattern of cross-domain statistical associations linking electrophysiological and kinematic indicators. Using graph-based correlation-network reconstructions within the Network Physiology framework (Bashan et al., 2012; Ivanov, 2021), we found that by 28 dpi the SCI + TMT group showed a correlation-network structure that was more similar to the intact pattern than that observed in untreated SCI animals. Importantly, these networks describe statistical associations between summary indicators and should not be interpreted as direct evidence of causal physiological interactions.

### 4.1 Task-dependent CPG engagement: rhythm generation vs. pattern formation

The complementary use of forced treadmill stepping and voluntary open-field locomotion unmasked a dissociation between locomotor speed and rhythm regularity that can be interpreted within the two-level architecture of the CPG (McCrea and Rybak, 2008; Rybak et al., 2015; Rybak et al., 2006a; Rybak et al., 2006b).

On the treadmill, step duration converged to identically prolonged values in both groups by the end of the observation period, regardless of training status. Because the fundamental oscillation frequency of the rhythm-generating (RG) layer is dictated by tonic descending brainstem drive (Ausborn et al., 2018; Danner et al., 2017), this persistent slowing is consistent with the permanent structural loss of reticulospinal projections in the ventrolateral funiculus (Ballermann and Fouad, 2006; Schucht et al., 2002), which afferent feedback alone cannot fully restore in terms of cycle frequency, although it can sustain the act of stepping itself (Markin et al., 2010). This interpretation is reinforced by data from spinal cats, where the re-expression of locomotion and its speed modulation depend critically on the return of sufficient excitability within sublesional spinal circuits rather than on task-specific reorganization (Harnie et al., 2019), and by computational modeling showing that recovered animals retain reduced stepping frequency even after substantial circuit reorganization (Shevtsova et al., 2026).

However, the fixed belt speed masked critical training-induced enhancements in voluntary motor control. Voluntary overground locomotion requires active recruitment of the descending locomotor command system, from glutamatergic mesencephalic locomotor region (MLR) neurons in the cuneiform nucleus (CnF) and pedunculopontine nucleus (PPN) (Caggiano et al., 2018) through reticulospinal relay neurons in the caudal brainstem (Capelli et al., 2017), together with parallel monoaminergic drives (Jordan et al., 2008), whereas treadmill stepping can be maintained by peripheral feedback alone given sufficient baseline spinal excitability. The substantially greater recovery of overground stride time in the trained group therefore suggests that TMT may potentiate the functional gain of residual descending transmission for volitional stepping, likely through propriospinal relay circuits that are sufficient for bulbospinal command transmission (Cowley et al., 2010) and amenable to neurochemical facilitation (Zaporozhets et al., 2011), combined with repeated engagement of spinal monoaminergic neuromodulation during locomotor training (Noga et al., 2017).

Crucially, while maximal stepping frequency remained constrained by the structural ceiling on RG-layer drive, metrics reflecting the pattern formation (PF) network showed profound, training-specific normalization. The trained group produced more organized, continuous step cycles. Given comparable average step durations, this difference most likely reflects fewer cycle interruptions rather than a higher intrinsic stepping frequency. This pattern is consistent with prior work showing that trained contused rats restore normal step trajectories while untrained animals develop aberrant swing patterns (Heng and de Leon, 2009). A plausible mechanism is that load-related and hip-extension afferents activated repetitively during treadmill stepping engage polysynaptic excitatory pathways onto CPG rhythm-generating networks (Rossignol et al., 2006). Proprioceptive afferents below the lesion are required not only for initiating but also for maintaining locomotor recovery, as their ablation permanently reverts functional gains even in the presence of reorganized descending detour circuits (Takeoka and Arber, 2019). The involvement of intraspinal plasticity is supported by previous findings that trained contused rats retained improved kinematic parameters immediately after complete transection, indicating that BWSTT in the presence of spared descending pathways induces neuroplasticity at the lumbar spinal level (Singh et al., 2011). However, discontinuation of training after the transection abolished these kinematic gains within 2 weeks, indicating that continued training-driven sensory input is required to maintain spinal circuit reorganization.

Open-field stride time variability (Kvar) provided the most dramatic illustration of PF-layer recovery. In the two-level CPG framework, non-resetting deletions of motoneuron activity arise from disturbances within the pattern formation (PF) layer rather than the rhythm generator (Lafreniere-Roula and McCrea, 2005; Zhong et al., 2012). By analogy, we suggest that the elevated step-to-step variability characteristic of untreated post-SCI animals similarly reflects PF-layer instability, whereas the near-complete normalization of Kvar achieved by the trained group suggests that repetitive proprioceptive loading may help restore inhibitory precision within local spinal circuits, potentially through mechanisms such as upregulation of potassium-chloride cotransporter 2 (KCC2)-mediated chloride homeostasis via the brain-derived neurotrophic factor (BDNF)-KCC2 pathway (Beverungen et al., 2020; Cote et al., 2014), normalization of reciprocal flexor–extensor alternation (Bilchak et al., 2025), and recovery of presynaptic inhibition and phase-dependent afferent gating (Smith and Knikou, 2016). Given that stride-time variability is influenced by walking speed, as demonstrated in human gait studies (Beauchet et al., 2009), this recovery of regularity should be interpreted as improved PF-layer function despite the residual RG-layer slowing discussed above. To our knowledge, the explicit mapping of the speed–regularity dissociation onto RG versus PF layers of the two-level CPG has not been previously articulated in the SCI rehabilitation context and represents a novel interpretive contribution of this study. This selective recovery of stepping regularity and continuity, rather than maximal speed, may contribute to the stronger cross-domain association pattern observed in the trained group, where electrophysiological and kinematic nodes form a correlation-network structure closer to the intact pattern.

### 4.2 Postural calibration and the canalization of compensatory strategies

Kinematic analysis revealed that functional recovery operates via compensation around a shifted physiological baseline. While dynamic range of motion (ROM) normalized comparably in both groups by the end of the observation period, absolute joint angles remained persistently shifted toward extension. This dissociation suggests that the capacity for reciprocal alternation between flexor and extensor half-centers, which contributes to the dynamic excursion of limb movement (Ausborn et al., 2018; Latash et al., 2020), may recover independently of the tonic bias in joint position. The latter likely reflects the altered balance of residual descending drive to extensor motoneuron pools following incomplete SCI (Ballermann and Fouad, 2006), as reticulospinal plasticity has been shown to correlate with locomotor recovery after spinal hemisection. Computational models of spinal CPG circuits further demonstrate that the magnitude and distribution of brainstem drive to rhythm-generating centers is a key determinant of locomotor pattern output (Danner et al., 2016; Shevtsova et al., 2015), supporting the view that changes in descending input after injury would shift the operating point of the locomotor network. A conceptually analogous phenomenon has been described in cats with partial spinal cord injuries, where hindlimb locomotor patterns recover through CPG plasticity and compensatory mechanisms, yet persistent kinematic asymmetries and postural deficits remain, particularly on the side of the lesion (Barrière et al., 2008; Rossignol et al., 2009).

Importantly, the two groups followed divergent compensatory trajectories. Without structured afferent input, the injured spinal cord enters an unstable state where inter-individual variations in spared white matter (Jeffery et al., 2020; Loy et al., 2002) and differences in reticulospinal sprouting (May et al., 2017) and propriospinal relay reorganization (Courtine et al., 2008) allow untreated animals to develop highly variable compensatory strategies, characterized by extensor deficits and abnormal stepping patterns (Collazos-Castro et al., 2006; Heng and de Leon, 2009). In contrast, treadmill locomotion engages stance-phase proprioceptive feedback that reinforces extensor-loading patterns through load-sensitive afferent pathways (Frigon, 2017; Rossignol et al., 2006), potentially providing a more structured sensory environment compared to spontaneous open-field locomotion. Although basic re-expression of stepping may not require task-specific training after complete spinal transection (Harnie et al., 2019), structured treadmill input may shape the quality, regularity, and inter-individual consistency of recovery after incomplete SCI. The transient step height overshoot observed exclusively in the trained group may reflect an activity-dependent recalibration of proprioceptive synaptic input to flexor premotor circuits, consistent with the spontaneous rearrangement of proprioceptive afferent connectivity to flexor premotor interneurons demonstrated after spinal hemisection (Takeoka and Arber, 2019). Treadmill-driven repetitive loading is known to induce progressive plasticity in spinal sensorimotor pathways, including enhanced polysynaptic group I excitation in extensors (Rossignol et al., 2006), and could similarly amplify proprioceptive pathway reorganization in flexor circuits before the system reaches a steady state. That both groups ultimately recovered step height comparably indicates effective compensation by flexor burst-generating CPG elements even with limited residual supraspinal drive (Rossignol and Frigon, 2011). Ultimately, the structured sensory input of the treadmill appears to act as a canalizing force (Ferguson et al., 2012), channeling activity-dependent plasticity toward functionally relevant configurations and minimizing maladaptive plasticity that may develop in the absence of structured rehabilitation (Grau et al., 2017; Huie et al., 2017), thereby reducing the inter-individual variability observed in untreated cohorts. These kinematic data caution that conventional ROM metrics alone may overestimate the degree of functional normalization. The canalization of compensatory strategies toward stable, reproducible locomotor solutions may also explain why the trained group exhibits a more coherent cross-domain correlation structure, despite persistent shifts in absolute joint angles relative to the intact baseline.

### 4.3 Re-establishing interlimb coordination via propriospinal reorganization

The stabilization of local lumbar circuits was paralleled by the reintegration of cervico-lumbar coordination. Contusive injury to the ventrolateral funiculus partially severs long propriospinal neurons (LPNs) that interconnect the cervical and lumbar enlargements (Reed et al., 2006). Among these, genetically defined ascending V3 populations have been shown in mice to provide the diagonal RG-to-RG coupling essential for trot-like coordination (Zhang et al., 2022), while descending cervico-lumbar projection neurons of V0 and V2 identity contribute to interlimb coordination and postural stability (Ruder et al., 2016). That these connections operate at the RG level implies that disrupted fore-hind coordination after contusion reflects uncoupled rhythm generators rather than PF dysfunction, consistent with the anatomical vulnerability of inter-enlargement pathways and the corrupted temporal signaling through partially damaged propriospinal routes. Indeed, Shepard et al. (2021) and Shepard et al. (2023) demonstrated that spared long ascending propriospinal neurons (LAPNs) and long descending propriospinal neurons (LDPNs) after thoracic contusion carry temporally imprecise information that, rather than aiding recovery, actively interferes with lumbar stepping, suggesting that the coordination deficits observed in the untreated group may reflect both the loss and the dysfunction of surviving inter-enlargement neurons. In contrast, left-right alternation, mediated by commissural interneurons crossing within each segmental level below the lesion (Kiehn, 2016; Talpalar et al., 2013), was relatively preserved.

Structured quadrupedal TMT was associated with fore-hind temporal coordination closer to intact levels. Forced, synchronized quadrupedal stepping provides the temporally correlated pre- and post-synaptic activity necessary for Hebbian strengthening of bridging inter-enlargement LPN synapses (Bareyre et al., 2004; Courtine et al., 2008) and reticulo-propriospinal detour pathways (Filli et al., 2014), as demonstrated directly by Shah et al. (2013). However, active supraspinal engagement appears critical for maximizing translesional plasticity (van den Brand et al., 2012), an important caveat given that our treadmill protocol involves externally driven rather than volitionally initiated locomotion. The observation of a rostro-caudal severity gradient in stride time disruption is consistent with evidence that thoracic SCI can transiently alter forelimb somatosensory processing at the cortical level (All et al., 2021) and impair forelimb motor skill learning (Salimi et al., 2021), indicating that sensorimotor perturbations extend beyond the sub-lesional segments. The relatively spared cervical networks may serve as an intact functional anchor guiding temporal re-entrainment of the lumbar CPG.

### 4.4 Network-level interpretation of electrophysiology–kinematics associations

The correlation-network analysis provides an integrative view of how electrophysiological and kinematic indicators covary across animals during recovery. This approach should be interpreted as descriptive and hypothesis-generating: it identifies organized statistical relationships between summary indicators, but it does not by itself establish causal interactions between physiological subsystems.

After correction for multiple edge-wise comparisons, the surviving associations were sparse and therefore require cautious interpretation. The corrected association between left and right H-wave amplitudes may indicate a more symmetrical bilateral pattern of reflex-related electrophysiological variability in the trained group. This observation is consistent with the known importance of left–right spinal coordination during locomotion and with the role of commissural circuits in interlimb alternation (Kiehn, 2016; Talpalar et al., 2013). However, the present correlation analysis does not identify the underlying cellular pathway and should not be interpreted as direct evidence of restored commissural connectivity, normalization of presynaptic inhibition, or BDNF–KCC2-mediated chloride homeostasis. Rather, the bilateral H-wave amplitude association suggests that treadmill rehabilitation was accompanied by a more organized pattern of segmental reflex-related variability.

A second corrected association linked left H-wave latency with the median stride interval of the right hind paw. Because this is a contralateral electrophysiology–kinematics association, it is compatible with the hypothesis that treadmill training may improve the alignment between reflex-related timing and interlimb gait organization. At the same time, this finding should not be interpreted as proof of temporal coupling or causal reintegration of reflex arcs into locomotor pattern generators. The variables used in the network are scalar summary indicators rather than time-resolved physiological signals. Therefore, this association is best viewed as an exploratory marker of cross-domain covariation that may reflect, but does not directly demonstrate, partial reorganization of spinal reflex–gait relationships.

The corrected kinematic associations were concentrated mainly among hindlimb and knee stride-interval indicators. This pattern is consistent with the anatomical level of injury and the rehabilitation paradigm: thoracic contusion affects descending control of lumbar locomotor networks, whereas treadmill training repeatedly engages hindlimb loading and proprioceptive feedback pathways. The presence of corrected LHP–RHP and left knee–right knee associations may therefore reflect a more coherent bilateral hindlimb timing structure after rehabilitation. However, these associations do not prove normalization of left–right alternation at the circuit level. They indicate that treadmill training was associated with a more organized statistical relationship among hindlimb timing indicators.

The weaker involvement of forelimb-related indicators, including the absence of corrected RFP-related links, suggests that recovery of cervico-lumbar coordination may remain incomplete or asymmetric. This interpretation is compatible with previous evidence that long propriospinal pathways connecting cervical and lumbar enlargements are vulnerable after thoracic spinal cord injury and that spared pathways may transmit temporally imprecise information during recovery (Reed et al., 2006; Shepard et al., 2021; Shepard et al., 2023). However, the present dataset cannot determine whether the observed asymmetry reflects pathway-specific plasticity, residual lesion heterogeneity, or limited statistical power.

Finally, no corrected association involving M-wave amplitude or latency was detected. Because M-wave parameters mainly reflect peripheral neuromuscular activation, whereas H-wave parameters are more closely related to segmental reflex excitability, this finding provides a useful internal comparison. It suggests that the corrected association pattern was not driven primarily by generalized changes in peripheral neuromuscular transmission. Nevertheless, the absence of M-wave associations should not be treated as a formal negative control, and the H-wave-related findings remain exploratory. Overall, the corrected network results support the interpretation that treadmill rehabilitation is associated with a more organized pattern of reflex-related and hindlimb kinematic associations, while direct mechanistic confirmation will require longitudinal electrophysiological recordings, perturbation experiments, or time-resolved analyses during stepping.

### 4.5 Limitations

Several methodological constraints should be acknowledged. The study was performed in a relatively small final cohort (n = 18; n = 9 per group), with the number of valid observations varying across tests, parameters, and time points. This limits statistical power particularly for correlation-network analysis. The use of only female Wistar rats restricts generalizability given well-documented sex-dependent differences in neuroinflammatory responses and locomotor recovery trajectories after SCI. The treadmill protocol involves externally driven locomotion, which may limit the degree of supraspinal engagement compared to motivation-driven paradigms; notably, Harnie et al. (2019) demonstrated locomotor recovery without task-specific training, suggesting that structured TMT may primarily accelerate and standardize rather than enable recovery. The correlation-network analysis captures pairwise associations between summary indicators without establishing causal directionality, and the absence of individual-level white matter sparing quantification precludes direct correlation between anatomical damage extent and network recovery metrics. Finally, the 28-day observation window and the use of a single injury severity limit conclusions about chronic outcomes and dose–response relationships across the SCI severity spectrum.

### 4.6 Concluding remarks

Moving beyond single-metric assessments, this study suggests that treadmill rehabilitation after SCI is associated with both improvement in selected locomotor metrics and partial reorganization of cross-domain statistical association patterns. Convergent data from fixed-speed and voluntary locomotor environments indicate that treadmill training may preferentially improve the regularity and coordination of stepping, while absolute locomotor frequency remains constrained after injury. Correlation-network reconstruction provides an additional descriptive layer by showing how electrophysiological and kinematic indicators covary across animals during recovery. These findings support the use of network-based summaries as complementary, hypothesis-generating tools in preclinical SCI rehabilitation studies. Future work should test whether longitudinal network metrics, such as network density, clustering coefficient, or modularity, can predict rehabilitation outcomes and whether experimentally targeted perturbations can establish causal links between reflex modulation, gait organization, and functional recovery.

## Data Availability

The source code used for the analysis is available at https://github.com/Griga21/Moving-Composite-Analysis. The original contributions presented in the study are included in the article/Supplementary Material; further inquiries can be directed to the corresponding authors.
